# Effects of Velvet Antler with Blood on Bone in Ovariectomized Rats

**DOI:** 10.3390/molecules170910574

**Published:** 2012-09-05

**Authors:** Sung-Hui Tseng, Hsin-Ching Sung, Lih-Geeng Chen, Ying-Jang Lai, Kun-Teng Wang, Chun-Hsien Sung, Ching-Chiung Wang

**Affiliations:** 1 School of Medicine, College of Medicine, Taipei Medical University, 250 Wu-Hsing Street, Taipei City 11031, Taiwan; Email: m003089010@tmu.edu.tw; 2 Orthopedics Research Center, Taipei Medical University Hospital, 252 Wu-Hsing Street, Taipei City 11031, Taiwan; 3 Department of Anatomy, Chang Gung University, 259 Wen-Hwa 1st Road, Kwei-Shan Township, Taoyuan County 33302, Taiwan; Email: tim0603kimo@yahoo.com.tw; 4 Department of Microbiology, Immunology and Biopharmaceuticals, College of Life Sciences, National Chiayi University, No. 300 Syuefu Rd., Chiayi City 60004, Taiwan; Email: lgchen@mail.ncyu.edu.tw; 5 Department of Food Science, National Quemoy University, No.1, Daxue Rd., Jinning Township, Kinmen County 89250, Taiwan; Email: lai@kmit.edu.tw; 6 School of Pharmacy, College of Pharmacy, Taipei Medical University, 250 Wu-Hsing Street, Taipei City 11031, Taiwan; Email: b8706014@tmu.edu.tw; 7 Graduate Institute of Pharmacognosy, College of Pharmacy, Taipei Medical University, 250 Wu-Hsing Street, Taipei City 11031, Taiwan; Email: chsung@fda.gov.tw

**Keywords:** velvet antler, velvet antler blood, velvet antler middle section, osteoporosis, ovariectomized animal model

## Abstract

In traditional Chinese medicine (TCM), both velvet antlers (VA) and VA blood can tonify qi, essence, and marrow, nourish the blood, and invigorate bones and tendons. In TCM, the combination of VA and VA blood is believed to have superior pharmacological effects. Scientific evidence supporting the traditional therapeutic preference for redder antler is needed. The effectiveness of the combination therapy of VA middle sections (VAMs) and VA blood (VAM-B) was first examined in promoting proliferation of mouse osteoblastic cells (MC3T3-E1). The anti-osteoporotic activity of VAM-B (ratio of VAM:VA blood = 1:0.2) was evaluated with ovariectomized (OVX) rats at a dose of 0.2 g/kg. In VAM-B-treated OVX rats, the body weight decreased 10.7%, and the strength of vertebrae and the femur respectively increased 18.1% and 15.4%, compared to the control. VAM-B treatment also recovered the estrogen-related loss of the right tibial trabecular bone microarchitecture. Alkaline phosphatase (ALP) significantly decreased, but estradiol did not significantly change in serum of VAM-B-treated OVX rats. We also provide an effective strategy to enhance the anti-osteoporotic activity of VAM. In conclusion, our results provide scientific evidence supporting the traditional therapeutic preference of redder antler and indicate that VAM-B is a potential therapeutic agent for managing osteoporosis.

## 1. Introduction

According to the *WHO International Standard Terminologies on Traditional Medicine in the Western Pacific Region* published in 2007, the kidney in traditional Chinese Medicine (TCM) is the viscera which stores vital essence, and promotes growth, development, reproduction, and urinary function [1]. This viscera is also responsible for the functions and activities of bones and marrow. A TCM classic states that when a woman is in the menopausal year, the kidney qi is insufficient, and the kidney yin and yang become imbalanced [2]. Restoring kidney qi may alleviate related symptoms, including osteoporosis. Previous experiments conducted with normal mice or ovariectomized animals demonstrated the efficacy of several kidney yang-reinforcing herbs in increasing bone density [3,4].

Deer products like velvet antlers (VAs) or VA blood are also classified as kidney yang-tonifying and -replenishing medicines in *Bencao Gangmu* (Compendium of Materia Medica), a famous TCM classic written by Li Shi-Zhen about 500 years ago. Therapeutically, both VA and VA blood tonify qi, essence, and marrow, nourish the blood, and invigorate bones and tendons. A previous study reported that long-term antler administration (13 months) moderated decreased plasma phosphorus and calcitonin levels and femoral bone density and calcium content, and increased plasma parathyroid hormone (PTH) and alkaline phosphates (ALP) activity levels associated with an ovariectomy (OVX) in 2 month-old senescence-accelerated mouse (prone-8, SAMP8) [5]. Others also demonstrated the protective effects of antler extracts or antler collagen on bones in either corticosteroid or OVX-induced animal models [6,7]. As for VA blood, investigators showed that daily administration of VA blood (4,000 μL/kg) for 10 weeks moderated the ovariectomy-induced reductions in the bone mineral density (BMD) of the lumbar spine and femur, and serum levels of insulin-like growth factor (IGF)-l and testosterone [8]. Taken together, the above data support the traditional claims that both VA and VA blood can modulate the functions and activities of bones, while they also support the future development of antler extracts for osteoporosis treatment.

TCM herbalists believe that the combination of VA and VA blood has superior pharmacological effects. Investigators demonstrated that the upper and upper mid-sections of VAs are generally darker and redder than either the tips or lower sections [9]. In an attempt to provide scientific evidence supporting the traditional therapeutic preference for redder antler and to develop an effective strategy to enhance the anti-osteoporotic activity of the lower portion of VA, a combination regimen comprising different portions of VA and VA blood was tested for its efficacy in moderating osteoporosis in an estrogen-deficient state and in stimulating the proliferation of osteoblastic cells.

## 2. Results

### 2.1. Combination Therapy of VAM and VA Blood is Effective in Inhibiting Osteoblast-Like Cells

We postulated that combination therapy of VA and VA blood would enhance the anti-osteoporotic activity of the lower portion of VA. In order to test our hypothesis, we first evaluated the interactions of the combination of VA upper sections (VAUs), VA middle sections (VAMs), and VA basal sections (VABs) with VA blood on the proliferation of MC3T3-E1 cells. The combined effect of VA and VA blood was evaluated using a combination index (CI), which was calculated using Calcusyn V2 software. A CI value of <1.0 indicated a synergistic interaction between VAM and VA blood at most of the dose combinations tested (Table 1). Since VAM and VA blood revealed synergistic interactions, subsequent studies were performed with the extracts that contained VAM and VA blood in a ratio of 1:0.2; this extract was abbreviated VAM-B.

**Table 1:** Synergistic analysis of the combination therapy with the upper (VAU), middle (VAM), and basal portions of velvet antler (VA) (VAB) and VA blood in MC3T3-E1 cells. ED, effective dose.

VA:VA Blood = 1:0.2	Combination Index (CI)		
	ED_50_	ED_75_	ED_90_
VAU	5.76 × 10^3^	7.63 × 10^5^	1.05 × 10^8^
VAM	0.97	0.74	0.55
VAB	3.12	6.42	13.14

### 2.2. Chemical Constituents

Content of proteoglycan, protein, cholesterol, testosterone, estradiol, IGF-1, calcium, and iron of VAM-B were analyzed. As shown in Table 2, VAM-B contained testosterone, estradiol, and IGF-1. The composition of amino acids in VAM-B is presented in Table 3. The top three amino acids in VAM-B were in the order glutamic acid, glycine, and aspartic acid. The presence of various chemical constituents suggested that the combinations of most of the ingredients in the VAM-B contributed to the activity of VA products.

### 2.3. Body Weight and Serum Markers

**Figure 1:**
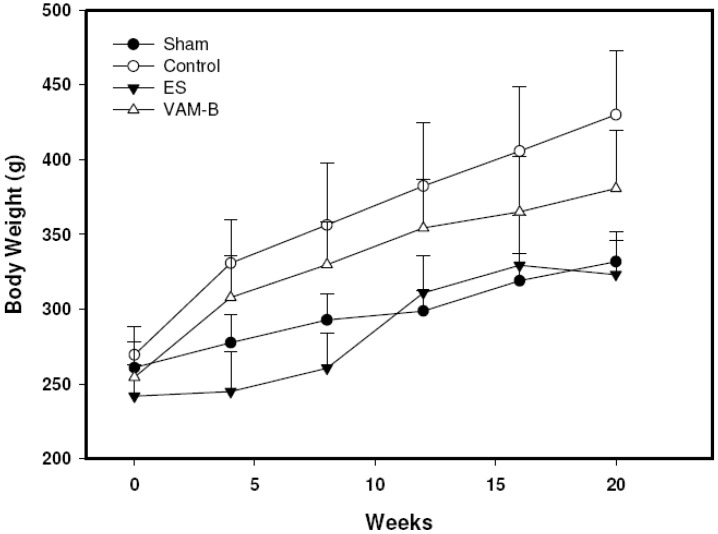
Body weight changes in sham, control (ovariectomized (OVX)-untreated), ethinyl estradiol (ES) (OVX+ES), andmiddle portion of velvet antler and blood combination (VAM-B) (OVX+VA-B) rats. Values are the mean ± SD, *n* = 8.

**Figure 2:**
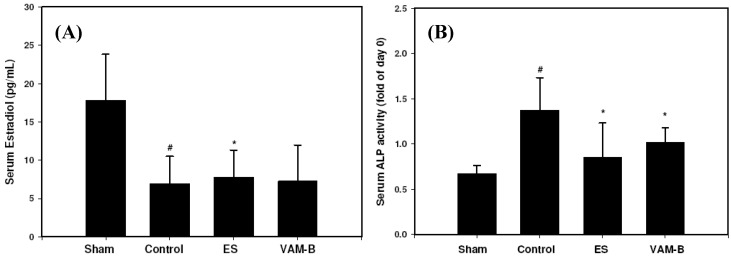
Serum estradiol (**A**), alkaline phosphatase (ALP) activity (**B**) in sham, control (ovariectomized (OVX)), ethinyl estradiol (ES) (OVX+ES), andmiddle portion of velvet antler and blood combination (VAM-B) (OVX+VA-B) rats at the end of the 20-week treatment. Values are mean ± SD, *n* = 8. ^#^ Compared to the sham group, *p* < 0.05; * compared to the control group, *p* < 0.05.

Body weights of all animals were measured every four weeks. Levels of serum ALP were measured at the initial and end points. Serum estradiol was measured at the end of the experiment. OVX-induced weight changes were moderated by oral administration of ethinylestradiol (ES) and VAM-B from the 4th week to the end of the experiment, suggesting that VAM-B has estrogen-like effects against the weight gain associated with estrogen deficiency (Figure 1). OVX-untreated rats showed a significantly lower level of serum estradiol compared to the sham group. Treatment with ES at 0.1 mg/kg/day but not with VAM-B significantly moderated the reduction (Figure 2A). OVX-untreated rats showed a significant increase in serum ALP activity compared to the sham group. ALP levels of both the OVX+ES and OVX+VAM-B groups significantly decreased compared to the OVX group (Figure 2B).

**Table 2:** Chemical analysis of the combination of the middle portion of velvet antler and velvet antler blood (VAM-B).

Item	VAM-B
Protein (mg/g)	0.5
Glycoprotein (mg/g)	3.3
Testosterone (ng/g)	4.4
Estradiol (ng/g)	3.1
Insulin-like growth factor-1 (ng/g)	33.8
Cholesterol (mg/g)	0.9
Calcium (%)	13.2
Iron (ppm)	434.0

**Table 3:** Analysis of amino acids in the middle portion of velvet antler combined with velvet antler blood (VAM-B).

Amino acid (mg/g)	VAM-B
Alanine	14.45
Arginine	15.95
Aspartic acid	18.21
Cysteine	1.73
Glutamic acid	21.00
Glycine	18.86
Histidine	6.51
Isoleucine	2.90
Leucine	15.66
Lysine	10.84
Methionine	2.11
Phenylalanine	8.72
Proline	12.78
Serine	8.39
Threonine	8.24
Tyrosine	4.00
Valine	10.01
**Total amounts**	**180.39**

### 2.4. Bone Biomechanics and Histomorphometric Parameters

**Figure 3:**
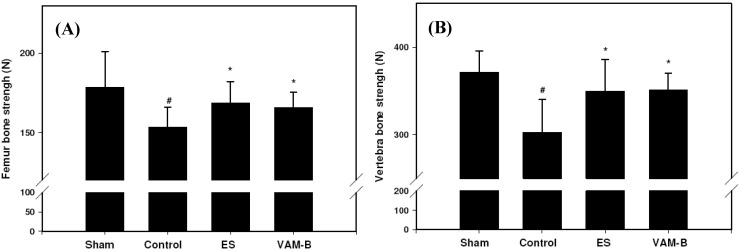
Maximal load values of the femur (**A**) and first lumbar vertebra (**B**) in sham-operated, control (ovariectomized (OVX)), ethinyl estradiol (ES) (OVX+ES), andmiddle portion velvet antler and blood combination (VAM-B) (OVX+VA-B) rats at the end of the 20-week treatment. Values are the mean ± SD, *n* = 8. ^#^ Compared to the sham group, *p* < 0.05; * compared to the control group, *p* < 0.05.

**Figure 4:**
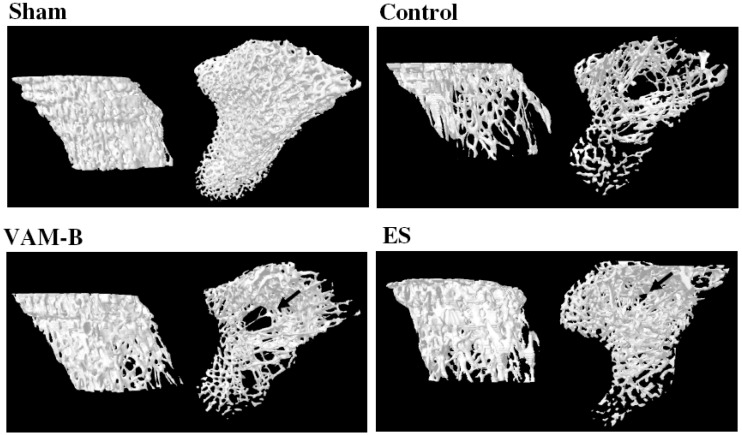
Microarchitecture of the tibia bone in sham-operated, control (ovariectomized (OVX)), ethinyl estradiol (ES) (OVX+ES), andmiddle portion velvet antler and blood combination (VAM-B) (OVX+VA-B) rats at the end of the 20-week treatment. The arrow indicates loss of the trabecular bone structure.

The strengths of both the femur and vertebral bone were significantly reduced in rats after OVX compared to sham-operated rats. OVX-induced reduction in the load values of femur were significantly reversed by the administration of ES or VAM-B compared to OVX untreated rats (Figure 3A). Strengths of the femur and vertebrae respectively increased 9% and 16% in VAM-B-treated OVX rats compared to the control group. Similarly, the loss of strength of the first vertebra was significantly reversed by ES and VAM-B treatment (Figure 3B). Assessment of the tibia cancellous bone architecture was performed with micro-CT, and histomorphometric parameters were calculated. Figure 4 shows that VAM-B and ES treatment recovered the trabecular bone structure of the tibia in ovariectomized rats. OVX induced significant decreases in the bone volume (BV/TV) and trabecular number (Tb.N), and increases in trabecular separation (Tb.Sp) and the structure model index (SMI) at week 20 compared to sham-operated rats, which indicates the loss of trabecular bone volume as well as disruption of the trabecular microarchitecture. Daily treatment with ES and VAM-B attenuated these changes (Table 4).

**Table 4:** Structural parameters of the proximal tibia of all groups from micro-CT.

Groups	BV/TV (%)	Tb.Th (μm)	Tb.Sp (μm)	Tb.N (μm)	SMI
Sham	40.84 ± 10.73	101.66 ± 16.09	168.28 ± 29.25	0.0040 ± 0.0004	0.60 ± 0.38
Control	10.70 ± 3.32 ^#^	107.69 ± 9.77	696.22 ± 158.76 ^#^	0.0010 ± 0.0003 ^#^	2.01 ± 0.22 ^#^
VAM-B	16.15 ± 2.23 *	105.60 ± 2.63	447.25 ± 65.28 *	0.0015 ± 0.0001 *	1.70 ± 0.10 ^#^
ES	25.69 ± 10.41 *	90.29 ± 4.01	255.99 ± 92.24 *	0.0028 ± 0.0010 *	1.29 ± 0.52 ^#^

^#^ Compared to the sham group, *p* < 0.05; * compared to the control group, *p* < 0.05. BV/TV, bone volume/total volume; Tb.Th, trabecular thickness; Tb.N, trabecular number; SMI, structure model index.

## 3. Discussion

The prevalence and associated health-related burden of osteoporosis are increasing as men and women tend to live longer [10]. Despite there being several effective osteoporosis medications, many investigators are still searching for more-acceptable and safer agents from natural sources to prevent and treat osteoporosis. VA, which is the immature antler of male deer, has been used in TCM for more than 2,000 years. In TCM, a whole VA stick is usually divided into four portions, with the value decreasing from the tip to the base [11]. One of the factors that determine the price of VA is whether the antler shows an evenly distributed red color throughout the core [9]. A study by Wang *et al*. reported that there were no differences in concentrations of total amino acids, total phospholipids, calcium, and phosphates between VA slices with or without blood [12]. In this study, we demonstrated that the therapeutic potency of the lower VA section increased synergistically when treatment was combined with VA blood. The results of our study suggest that VA and VA blood act synergistically to enhance the anti-osteoporotic activity of the lower portion of VA, which provides scientific evidence supporting the traditional therapeutic preference for redder antler.

In this study, we found that blood usually settles near the antler tip when it is inverted after removal. In order to increase the portion of the VA core with red color and therefore the value of VA, it is need to spend longer processing times, so that the remaining blood can be spread more evenly throughout the antler. In fact, investigators conducted strenuous experiments to determine the effects of various types of removal techniques and post-removal handling on VA color in order to compete with antler products from other countries [9]. The authors concluded that sedative drug treatments resulted in less-red VA, while placing the velvet at an angle of 15° (tip down) gave a darker and redder antler than the typical fully inverted position. The method we used to prepare VAM-B in our study may provide another alternative to obtain VA products containing blood.

What are the active ingredients in VAM-B which work against osteoporosis? At present, the answer is unclear. During bone remodeling, estrogens have a clear role in decreasing osteoclast formation and activity; in contrast, androgens have potent effects on osteoblast proliferation and differentiation [13]. Sexual hormones should be some of the important biological substances mediating the therapeutic activity specific to VAM-B in bone. A previous study showed that in the serum, antler bone, and velvet of white-tailed deer (*Odocoileus virginianus*), the level of testosterone in the serum was the highest, while estradiol concentrations in serum were higher in the antler bone and velvet than in the serum [14]. Others also reported that there was no significant difference in serum constituents between blood samples collected during the antler growth period in stags of sika deer (*C. nippon*), except the level of ALP which increased during antler growth [15]. Antlers grow when testosterone consistently rises from a low level [16,17]. The VAM-B used in this study was obtained 75 days after casting, the time when serum testosterone should be at a high level (data not shown). In *Bencao Gangmu*, the medicinal properties of deer blood are classified as sweet, salty, and hot, while those of VA are sweet, salty, and warm. The different concentrations of estrogen and testosterone in the serum and velvet may explain differences in the basic properties between deer blood and VA as perceived by ancient TCM herbalists. IGF-1 helps maintain the bone mass [18]. A previous study also reported a positive correlation between the BMD and IGF-1 levels in the lumbar (L1–4) and trochanteric regions of patients with fibromyalgia syndrome (FMS) [19]. Although the role of IGF-1 in antler growth has been disputed [18,20], the presence IGF-1 within VAM-B makes this product a potential therapeutic agent worth future development for managing osteoporosis. Together, this evidence suggests that the synergistic effect of VAM-B as a potential anti-osteoporotic agent may be related to the presence of therapeutic concentrations of testosterone, estradiol, and IGF-1.

An estrogen deficiency leads to high bone turnover, which then affects the quality and quantity of bone tissues. We used an objective qualitative measurement to demonstrate the beneficial effect brought about by VAM-B on the trabecular microarchitecture of OVX rats. VAM-B treatment was associated with an increase in the trabecular bone mass, and also with an increase in trabecular numbers and trabecular connectivity. In terms of bone turnover markers, VAM-B treatment significantly suppressed ALP activity in OVX rats, similar to that of estrogen. However, the level of serum estradiol did not significantly rise in our OVX animals. Indeed, the estrogen content in the VAM-B extract used in this experiment was 3.05 ng/g, which is much lower than the standard dosage of conjugated estrogen for postmenopausal osteoporosis (which is 0.625~1.25 mg daily). These results indicate that VAM-B could protect bone by moderating enhanced bone resorption due to estrogen deprivation. The results also suggested that VAM-B may have a lower risk of adverse effects associated with prolonged hormone therapy, which is one of the factors associated with non-adherence to osteoporosis therapies [21]. Different species of deer have different levels of estradiols in the velvet; Pere David’s deer (*Elephurus davidianus*) had a higher estradiol concentration than Sika deer or fallow deer (*Dama dama*) [22]. Therefore, the optimal content of hormones within the VAM-B product must be contemplated and defined. Since products from animals may carry potential risks to human health, VAM-B should be processed in facilities that adhere to quality-assurance manufacturing practices. Also, it is still necessary to identify the active components for development and to implement product standards that ensure product uniformity and encourage consumer confidence in the products.

## 4. Experimental

### 4.1. Preparations of VA with Blood Powder

Sika deer (*Cervus nippon*) used in this experiment were cared for according to the Ethical Regulations on Animal Research of Taipei Medical University (approval No.: LAC-99-0327). Fresh VAs were harvested from sika deer 75 days after casting. These VAs were then divided into upper (VAU), middle (VAM), and basal sections (VAB). Each section was sliced, dried, and ground into powder. The powdered VA was immersed in 60% ethanol for 1 month. The extract was then filtered and freeze-dried. Yields of the sections, from upper to basal, were 11.45%, 7.68%, and 5.45%, respectively. VA blood powder was made from the blood collected when the VA was harvested.

### 4.2. Cell Proliferation Assay and Combination Effect between VA and VA Blood

MC3T3-E1 cells (ATCC: CRL-2593, an osteoblast-like cell line from C57BL/6 mouse calvaria) were obtained from the American Type Culture Collection (Rockville, MD, USA). MC3T3-E1 cells were cultured at 37 °C in a 5% CO_2_ atmosphere in α-modified minimal essential medium (α-MEM; GIBCO). Unless otherwise specified, the medium contained 10% heat-inactivated fetal bovine serum (FBS), 100 U/mL penicillin, and 100 mg/mL streptomycin. Cells were treated with different doses of VAU, VAM, or VAB and VA blood. In this study, VA powder was mixed with 0.2-fold VA blood as test samples based on findings of our previous experiments. The doses of VAs and VA blood for combination were chosen in fixed-ratio increments. After 24 h, α-MEM (90 μL) and 3-(4,5-dimethylthiazol-2-yl)-2,5-diphenyltetrazolium bromide (MTT, 10 μL) were added to the wells of a 96-well plate for 4 h. Supernatants (50 μL) were taken from the wells, and isopropanol in a 0.04 N HCl solution (200 μL) was added. The absorbance of the resulting solution was measured at 600 nm with a μQuant spectrophotometer (BioTek, Winooski, VT, USA) to calculate cell proliferation rates.

The response to each treatment was utilized to perform a synergy analysis with Calcusyn V2 software (Biosoft, Cambridge, UK). Specifically, ED_50_, ED_75_, and ED_90_ doses of combination therapy of VAU, VAM, and VAB with VA blood respectively responsible for 50%, 75%, and 90% of the maximal proliferative effect were calculated using the combination index (CI). The definition used previously to define the combination effect was applied as follows: CI < 1 was a synergistic effect; CI = 1 was an additive effect; and CI > 1 was an antagonistic effect [23].

### 4.3. Protein Content

The protein content was determined by the Bradford method [24]. In brief, a serially diluted bovine serum albumin solution (0.1 mg/mL) was used as the protein standard solution and reacted with the Bradford solution. One milliliter of sample solution was mixed well with 1 mL of the Bradford solution and allowed to stand for 10 min. Quantitative analysis of protein was performed with an enzyme-linked immunosorbent assay (ELISA) reader at an absorption wavelength of 595 nm.

### 4.4. Estradiol, Testosterone, and IGF-1 Analyses

The sample solution (VAM-B, 0.1 g) was first ultrasonically extracted with 5 mL of water for 30 min and then partitioned with 25 mL ether, and the ether layer was concentrated. The estradiol, testosterone, and IGF-1 contents of the ether extract of VAM-B were respectively measured with 17β-estradiol, testosterone, and IGF-1 EIA kits (Enzo Life Sciences, Lausen, Switzerland).

### 4.5. Rat Osteoporosis Model

Sprague-Dawley (SD) rats used in this experiment were cared for according to the Ethical Regulations on Animal Research of Taipei Medical University (approval No.: LAC-99-0327). In this study, 12 week-old female SD rats were randomized into five groups with eight rats each. Rats were first anesthetized, and a bilateral ovariectomy (OVX) was performed. A sham operation was performed using the same procedure without removing the ovaries. The sham-operated and OVX groups were treated with 5 mL/kg/day distilled water. The positive control group was treated with 0.1 mg/kg/day ethinyl estradiol (ES). The experimental groups were treated with 0.2 g/kg/day extracts of VAM-B. The treatment began one day after the operation and continued for 20 weeks. Animals were weighed every four weeks.

### 4.6. Bone Breaking Strength Test

The first lumbar vertebra and femur were obtained immediately after the animals were sacrificed. The biomechanical strength of the femur was measured by a 3-point bending test and of vertebra with a Bone and Wound Breaking Strength Tester (Model TK-252C, Muromachi Kikai, Tokyo, Japan). A bone was placed horizontally on a 2-point sample holder (with a 16-mm span). A load was placed at the center of the bone at a rate of 10 mm/min until the bone broke. The breaking load was expressed in Newtons (N).

### 4.7. Micro-Computed Tomographic (Micro-CT) Analysis

We obtained bone morphometric parameters of the right tibia metaphysis, including the bone volume over the total volume (BV/TV), trabecular number (Tb.N), trabecular separation (Tb.Sp), and trabecular thickness (Tb.Th), after scanning the upper tibia metaphysis with SkyScan 1076 Micro-CT (Skyscan 1076; Skyscan, Antwerp, Belgium), and by accordingly analyzing the volume of interest. Micro-tomographic slices were acquired at 500 projections and reconstructed with a spatial nominal resolution of 35 μm. The voxel size was 35 μm. Files were imported into CTAn software (SkyScan) for 3D analysis and image generation. The operator conducting the scan analysis was blinded to the treatment associated with the specimens.

### 4.8. Statistical Analysis

Data were first statistically assessed by a one-way analysis of variance (ANOVA), followed by Scheffe’s test for comparison of means. *p* < 0.05 was considered statistically significant. All data are expressed as the mean ± S.D.

## 5. Conclusions

In conclusion, the presence of estrogen, testosterone, and IGF-1 and enriched protein contents within VAM-B makes this product a potential therapeutic agent worth future development for managing osteoporosis in women. The results showed the VA with blood had significant tonic effects. The results also provide scientific evidence supporting the traditional therapeutic preference for redder antler and provide an effective strategy to enhance the anti-osteoporotic activity of VAM.
